# A prospective analysis of adverse effects of antiretroviral agents: Additional points to consider

**DOI:** 10.4103/0253-7613.64491

**Published:** 2010-04

**Authors:** Megha Shah

**Affiliations:** Department of Pharmacology, Medical College, Vadodara, Gujarat

Sir,

I have read the article "A prospective observational cohort study to elicit adverse effects of antiretroviral agents in a remote resource-restricted tribal population of Chhattisgarh".[1] It was about assessment of adverse drug reactions (ADRs) to antiretroviral therapy. The article was informative and interesting. Certain clarifications would however make the article more informative. For example, it was found that ADRs to antiretroviral therapy are more common in patients with hepatitis.[2,3] Was hepatitis an exclusion criteria in the study? The number of patients or cases of ADRs is also not clear. Authors mentioned that a total of 120 ADRs were noted in 68 cases. It is also mentioned that out of 137 HIV patients, 16 stopped therapy midway; the reasons for the same however should also be mentioned. The outcome of severe reactions and causality assessment done may also be elaborated. From this study, it was concluded that severe ADRs to HAART are more common in females and those with CD4 counts less than 200 cells/mm^3^. It would help to clarify if there was any difference in average age and CD4 counts among males and females.
